# Progress and Challenges in Targeting BRAFV600E-mutant Colorectal Cancer: Molecular Pathogenesis, Treatment Strategies, and Future Directions - My Experience at Johns Hopkins University -

**DOI:** 10.14789/ejmj.JMJ25-0012-R

**Published:** 2025-08-30

**Authors:** HISASHI RO, SHUN ISHIYAMA, KIICHI SUGIMOTO, KAZUHIRO SAKAMOTO

**Affiliations:** 1 Department of Coloproctological Surgery, Juntendo University Faculty of Medicine, Tokyo, Japan; 1 Department of Coloproctological Surgery, Juntendo University Faculty of Medicine, Tokyo, Japan

**Keywords:** BRAFV600E, colorectal cancer, MAPK pathway, drug resistance, targeted therapy

## Abstract

Colorectal cancer (CRC) remains a major global health challenge, with the BRAFV600E mutation representing one of the most aggressive and treatment-resistant subtypes. This review focuses on the molecular pathogenesis, clinical implications, and evolving therapeutic strategies for BRAFV600E-mutant CRC. The BRAFV600E mutation leads to constitutive activation of the MAPK signaling pathway, driving tumor proliferation, immune evasion, and resistance to conventional therapies. Despite advances in targeted therapy, including the use of BRAF inhibitors, clinical responses are often short-lived due to feedback activation of EGFR and alternative survival pathways. Combination therapies, such as the BEACON CRC regimen (BRAF, MEK, and EGFR inhibitors), have shown improved outcomes, yet resistance remains a significant obstacle. Our research investigated the potential synergy between BRAF inhibitors, Wnt pathway inhibitors, and epigenetic modifiers, such as DNA methyltransferase (DNMT) inhibitors, in enhancing therapeutic efficacy. Preliminary results suggest that targeting both oncogenic signaling and epigenetic dysregulation may overcome resistance mechanisms in BRAFV600E-mutant CRC. Additionally, we highlight the role of circulating tumor DNA (ctDNA) as a promising biomarker for real-time monitoring of treatment response and clonal evolution. The integration of multi-targeted therapies and liquid biopsy technologies represents a critical step toward precision oncology. Understanding the interplay between genetic mutations, epigenetic alterations, and the tumor microenvironment is essential for developing more durable and personalized treatment strategies. This review outlines current advancements and future directions for managing BRAFV600E-mutant CRC, aiming to improve patient outcomes in this challenging disease subset.

## Introduction

**Figure 1:**
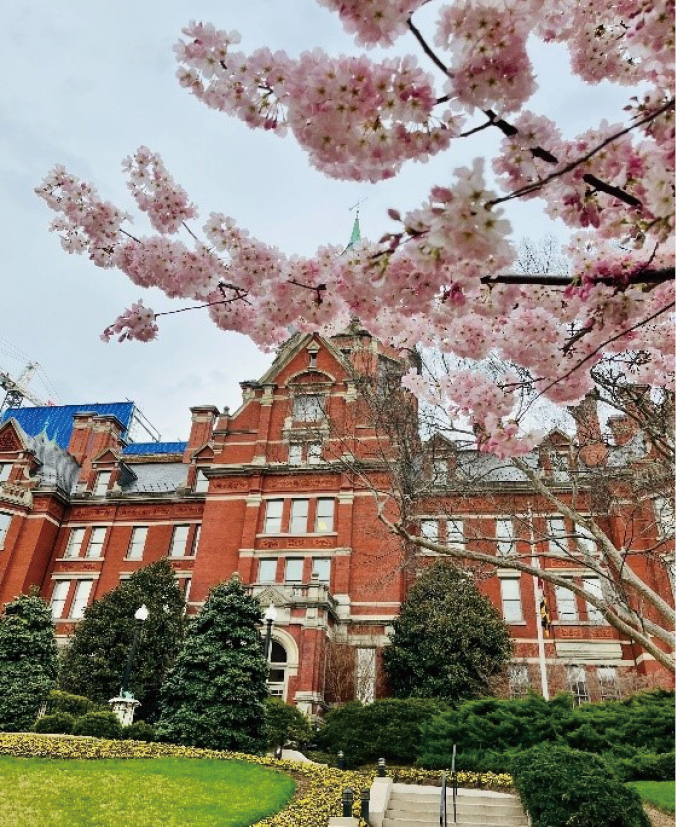
The appearance of the main building of Johns Hopkins University Hospital

**Figure 2:**
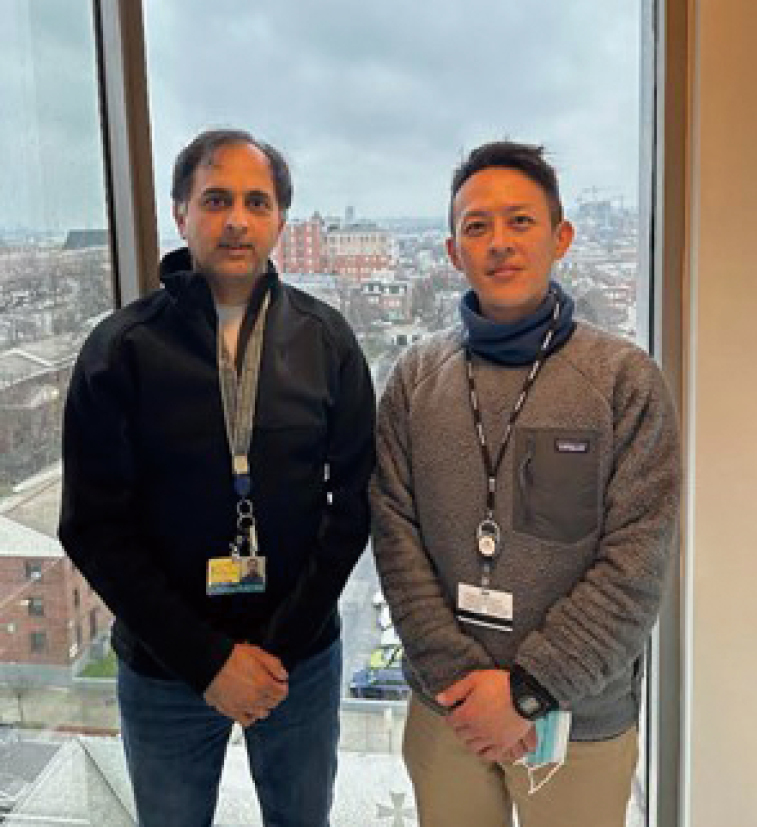
The photo with Associate Professor of Oncology Hariharan P. Easwaran

**Figure 3:**
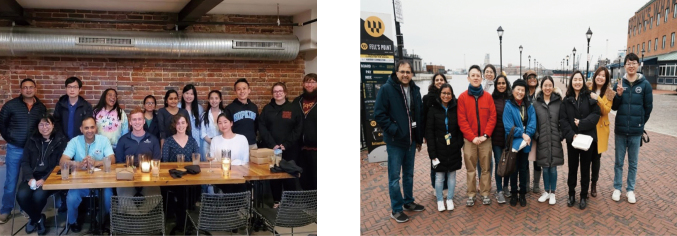
Lab members

I conducted research on the BRAFV600E mutation in colorectal cancer (CRC) at Johns Hopkins University in Maryland, United States, from April 2021 to March 2023 (Figure 1). I was conducting research in the laboratory of Hariharan P. Easwaran, who works under the supervision of Stephen B. Baylin, the Virginia and D.K. Ludwig Professor for Cancer Research and Professor of Oncology and Medicine at the Johns Hopkins University School of Medicine. I was assigned to work with Hariharan P. Easwaran, an associate professor of oncology at the Johns Hopkins University School of Medicine (Figure 2). He is affiliated with the Epigenetics and Cancer Biology Program at the Sidney Kimmel Comprehensive Cancer Center, where I also had the opportunity to conduct research (Figure 3). His research focuses on the interplay between epigenetics and cancer, particularly examining how DNA methylation patterns and histone modifications drive cancer development and progression. He is also working on epigenetics-based cancer therapies.

My research focused on how BRAFV600E CRCs rely on Wnt pathway activation. The key question was whether Wnt inhibitors could be used in combination with BRAF inhibitors. In this review, we explore the BRAFV600E mutation in CRC, covering its molecular pathogenesis, therapeutic approaches, and future directions.

CRC remains one of the most prevalent and deadly malignancies worldwide, ranking as the third most common cancer and the second leading cause of cancer-related deaths globally. Despite significant advances in screening techniques, early detection methods, and the development of new therapeutic strategies, CRC incidence and mortality rates have not shown significant decline. This indicates that significant obstacles remain in achieving effective treatment, particularly for certain molecular subtypes of CRC that exhibit more aggressive behavior and show resistance to standard therapies. Among these subtypes, the BRAFV600E mutation has emerged as one of the most clinically challenging and treatment-resistant variants. Understanding the pathogenesis, clinical implications, and potential treatment strategies for BRAFV600E- mutated CRC is crucial to improving patient outcomes and survival rates^1)^.

The BRAFV600E mutation involves the substitution of valine (V) with glutamic acid (E) at position 600 of the BRAF protein, a key component of the mitogen-activated protein kinase (MAPK) signaling pathway. This mutation leads to constitutive BRAF activation, which in turn triggers continuous downstream signaling pathways that regulate critical cellular functions, including proliferation, survival, and differentiation. In the context of CRC, this dysregulated signaling leads to uncontrolled growth of tumor cells, apoptosis evasion, and metastatic progression^2)^. As a consequence, BRAFV600E- mutant CRCs are typically more aggressive, showing poor differentiation and high rates of both peritoneal and distant organ metastases. Furthermore, this mutation is commonly linked to resistance against both standard chemotherapy and targeted therapies, making clinical patient management even more challenging^3)^.

Recent evidence indicates that the BRAFV600E mutation not only serves as a key driver of tumor progression but also shapes the tumor microenvironment, promoting immune evasion and therapeutic resistance. This mutation typically correlates with a highly immunosuppressive tumor microenvironment, marked by the recruitment of myeloid- derived suppressor cells (MDSCs) and regulatory T cells (Tregs), which inhibit antitumor immune responses. Understanding these interactions within the tumor microenvironment may reveal new avenues for immunotherapy-based treatment approaches.

The growing recognition of the BRAFV600E mutation as a key driver of aggressive CRC has sparked extensive research into the molecular mechanisms underlying its oncogenic effects.

Therefore, we aim to predict improved prognosis in BRAF V600E-positive CRC cells by combining Wnt inhibitors and epigenetic modifiers, specifically DNA methyltransferase (DNMT) inhibitors, alongside BRAF inhibitors, while investigating the associated factors.

We aimed to investigate whether Wnt inhibitors or epigenetic inhibitors could work synergistically with BRAF inhibitors to establish a novel therapeutic strategy for treating BRAF V600E-positive CRC.

## Materials and Methods

### Cell lines and culture conditions

Human CRC cell lines harboring the BRAFV600E mutation, including Col115, SW48, HT29, RKO, and NCI-H508, were used in this study. Among these, Col115, SW48, and HT29 also harbor APC mutations and serve as controls to evaluate whether Wnt inhibitors (Wnt-i) show enhanced efficacy in the absence of APC mutations. Additionally, CRC cell lines carrying APC mutations (SW480, DLD1) and those either harboring (SW480, DLD1) or lacking (HCT116) KRAS mutations were included for comparative analysis. All cell lines were sourced from authenticated cell banks and maintained in either RPMI-1640 or DMEM medium supplemented with 10% fetal bovine serum and 1% penicillin-streptomycin. Cells were incubated at 37°C in a humidified atmosphere containing 5% CO_2_.

### Ethical considerations

All human-derived cell lines used in this study were obtained from commercially available, authenticated cell banks. No research involving new human subjects was conducted. Therefore, this study does not fall under the category of human subject research and did not require approval from an Institutional Review Board (IRB) in accordance with the Declaration of Helsinki and other ethical guidelines. Nonetheless, all experiments were conducted in compliance with relevant institutional and international ethical standards.

### Drug treatments and combination studies

A panel of Wnt inhibitors (Wnt-i) targeting different levels of the Wnt signaling pathway is being tested both alone and in combination with the BRAF inhibitor vemurafenib. These inhibitors include compounds that block Wnt production/export (ICG1, IWPs), receptor/ligand interaction (OMP-18R5), Tankyrase (JW55), Dishevelled (CGP049090), β-Catenin/TCF interaction (NSC668036), and Wnt coactivators (ICG-001). The cells are exposed to varying concentrations of each inhibitor, both individually and in combination, and their effects on cell viability are assessed.

### Cell viability

Cell viability was assessed using the MTS assay to determine the half-maximal inhibitory concentration (IC50) for each drug.

### Epigenetic inhibitor assays

To investigate the role of epigenetic modifications in BRAFi response, selected cell lines are treated with the DNMT1 inhibitor GSK3685032 and the EZH2 inhibitor GSK126. The ability of these inhibitors to reactivate epigenetically silenced genes and enhance BRAFi sensitivity is evaluated through qRT-PCR and western blot analysis of key tumor suppressor genes.

### Study continuation

The ongoing research is being conducted by a postdoctoral fellow who took over the project following my departure from the laboratory.

## Molecular pathogenesis of BRAFV600E-mutant CRC

The BRAFV600E mutation plays a crucial role in CRC pathogenesis by driving aberrant signaling pathways that promote tumor initiation, growth, and treatment resistance. This mutation involves the substitution of valine (V) with glutamic acid (E) at codon 600 of the BRAF gene, a serine/threonine kinase that serves as an essential component of the MAPK signaling cascade. BRAF typically operates as part of a signaling pathway that relays extracellular growth signals from receptor tyrosine kinases (RTKs) to downstream components, including MEK1/2 and ERK1/2, ultimately regulating cellular processes such as proliferation, survival, and differentiation. However, when the BRAFV600E mutation is present, the BRAF kinase becomes constitutively activated, bypassing normal regulatory mechanisms and leading to continuous MAPK pathway stimulation. This leads to uncontrolled cell proliferation, resistance to programmed cell death, and enhanced tumorigenesis^3)^. Furthermore, recent studies have shown that BRAFV600E-mutant CRCs exhibit distinct molecular and histological features that set them apart from other CRC subtypes. These tumors are characterized by poor differentiation, mucinous histology, and a high likelihood of lymphovascular invasion, factors that contribute to their aggressive clinical behavior.

## MAPK pathway activation and tumorigenesis

The MAPK pathway plays a crucial role in multiple cancer-related processes, and its dysregulation is a hallmark of BRAFV600E-mutant CRC. In normal cells, MAPK pathway activation occurs through extracellular signals, typically mediated by RTKs, such as EGFR, which triggers RAS, a small GTPase protein. RAS activation then recruits RAF family kinases, including BRAF, to the plasma membrane, where BRAF phosphorylates MEK, leading to ERK activation. ERK translocates to the nucleus, where it regulates gene expression to drive cell cycle progression, survival, and differentiation. In cells harboring the BRAFV600E mutation, this signaling pathway becomes dysregulated due to constitutive BRAF activation, leading to continuous downstream signaling that occurs independently of receptor activation^4)^.

The BRAFV600E mutation leads to hyperactivation of BRAF kinase activity, resulting in sustained MEK1/2 and ERK1/2 phosphorylation. This sustained MEK-ERK signaling cascade activation plays a key role in driving cell proliferation and survival. In BRAFV600E-mutant CRC, this activation is typically accompanied by changes in the tumor microenvironment, including enhanced angiogenesis, immune evasion, and stromal remodeling, all of which promote tumor progression and metastasis.

It is worth noting that the sustained MAPK pathway activation in BRAFV600E-mutant tumors also triggers mechanisms that enable tumors to bypass normal regulatory checkpoints, including cell cycle checkpoints and programmed cell death (apoptosis). These tumors develop resistance to apoptosis through dysregulation of apoptotic proteins, including BCL-2 family members and p53, both of which are commonly altered in BRAFV600E- mutant cancers. Notably, recent studies have shown that concurrent mutations, particularly in TP53 and SMAD4, can further enhance MAPK hyperactivation, making these tumors even more refractory to conventional therapeutic approaches^5)^.

## Resistance mechanisms and feedback activation

While the MAPK pathway is a key driver in BRAFV600E-mutant CRC, targeting this pathway alone has significant limitations. A major challenge in targeting the BRAFV600E mutation is the rapid emergence of resistance. First-generation BRAF inhibitors, including vemurafenib and dabrafenib, showed promising preclinical efficacy but ultimately proved ineffective in the long term due to acquired resistance. This resistance largely stems from the activation of upstream signaling feedback mechanisms that counteract BRAF inhibition, including EGFR and HER2, which can reactivate the MAPK pathway.

In BRAFV600E-mutant CRC, this feedback loop presents a major challenge to the effective use of targeted therapies. Feedback activation occurs when BRAF inhibition leads to the upregulation of upstream signaling components (such as EGFR), which in turn reactivates the MAPK pathway. This compensatory mechanism enables tumor cells to survive and proliferate even in the presence of BRAF inhibitors. To address this challenge, combination therapies targeting multiple components of the MAPK pathway have been developed, such as combining BRAF inhibitors with MEK inhibitors (like binimetinib) or EGFR inhibitors (such as cetuximab)^6)^.

Emerging therapeutic strategies also focus on dual EGFR/HER2 inhibition, as recent studies indicate that simultaneously blocking these pathways may more effectively suppress resistance mechanisms.

**Figure 4:**
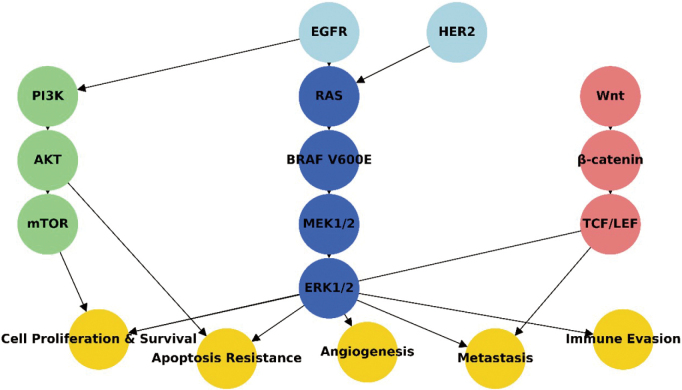
Signaling pathways involved in BRAFV600E-mutant colorectal cancer The MAPK pathway (blue) is persistently activated due to the BRAFV600E mutation, leading to uncontrolled cell proliferation and survival. The PI3K/AKT/mTOR pathway (green) contributes to apoptosis resistance and metabolic reprogramming, while the Wnt/β-catenin pathway (red) plays a role in tumor invasion and metastasis. Key downstream oncogenic processes include angiogenesis, immune evasion, and resistance to therapy (yellow). Targeted therapies for specific pathway components may improve treatment outcomes.

In addition to feedback activation, other signaling pathways, including PI3K/AKT and Wnt/β-catenin pathways, can also be upregulated in response to MAPK inhibition. These alternative pathways can contribute to drug resistance by promoting cell survival and proliferation despite MAPK pathway inhibition^7)^ (Figure 4).

## Epigenetic changes and CpG island methylator phenotype (CIMP)

Another critical feature of BRAFV600E-mutated CRC is its close relationship with epigenetic modifications, especially the CIMP. CIMP is characterized by widespread CpG island hypermethylation in tumor suppressor gene promoter regions, resulting in their silencing. In BRAFV600E-mutated CRC, the CIMP phenotype is frequently observed, contributing to tumorigenesis by deactivating key tumor suppressor genes, including MLH1, which plays a role in mismatch repair. MLH1 silencing leads to microsatellite instability (MSI), a condition that increases the genome-wide mutation rate and is associated with a more immunogenic tumor phenotype.

The presence of MSI, especially in CIMP-positive tumors, has important therapeutic implications. High MSI (MSI-H) tumors typically show enhanced response to immune checkpoint inhibitors, such as pembrolizumab and nivolumab, due to their higher mutational burden and increased antigenicity. However, only a subset of BRAFV600E- mutant CRCs are MSI-H, as many BRAFV600E- mutated tumors are microsatellite stable (MSS), which complicates immunotherapy decisions. In MSS tumors, immune evasion mechanisms, including the upregulation of immune checkpoint molecules (such as PD-L1), contribute to resistance against immunity-based therapies, which has driven research into combination therapies aimed at enhancing tumor immunogenicity. Furthermore, recent epigenomic studies indicate that CIMP-positive tumors may harbor distinct metabolic dependencies, revealing potential opportunities to target these vulnerabilities through metabolic inhibitors^8)^.

## Clinical and epidemiological features

BRAFV600E mutations are found in approximately 8-15% of all metastatic CRC cases, with a higher prevalence in right-sided colon cancers, elderly patients, and women. From a clinical perspective, these tumors typically exhibit distinct histological features, characterized by mucinous differentiation and poor tumor differentiation. These tumors are also characterized by high rates of peritoneal and distant organ metastases, particularly in the liver and lungs, contributing to poor clinical outcomes (Table 1). Recent studies have shown that the tumor microenvironment in BRAFV600E- mutated CRC exhibits distinct characteristics compared to other CRC subtypes, featuring elevated levels of TAMs and fibroblasts, which may explain its aggressive behavior. Understanding these interactions may reveal new therapeutic targets^9)^.

Patients with BRAFV600E-mutated CRC have a significantly worse prognosis compared to those with wild-type BRAF CRC. The median overall survival (OS) for metastatic BRAFV600E CRC typically ranges from 8 to 14 months, which is substantially lower than survival rates observed in patients with wild-type BRAF CRC. In addition to their aggressive behavior, BRAFV600E-mutant CRCs show resistance to standard chemotherapy regimens, including fluoropyrimidine-based treatments (such as 5-fluorouracil), oxaliplatin, and irinotecan. This chemotherapy resistance is attributed to sustained MAPK pathway activation and its downstream survival signaling. Given these clinical features, treatment strategies for BRAFV600E- mutated CRC should be tailored to the tumor’s specific molecular profile. Traditional chemotherapy regimens alone often prove inadequate, making it necessary to incorporate targeted alternative therapies to improve patient outcomes^3)^. Recent advances in liquid biopsy technologies, particularly the analysis of circulating tumor DNA (ctDNA), have enabled real-time monitoring of tumor progression and treatment response, paving the way for more personalized therapeutic approaches.

**Table 1:** Clinical and molecular characteristics of BRAFV600E-Mutant colorectal cancer

Category	Characteristic	BRAFV600E-Mutant CRC
Epidemiology	Prevalence in CRC	8-15% of metastatic CRC
	Gender distribution	Higher prevalence in females
	Common tumor location	Predominantly right-sided colon
Pathogenesis	Primary oncogenic driver	BRAFV600E mutation (V600E substitution)
	Key signaling pathways	Constitutive MAPK pathway activation (RAF-MEK-ERK cascade)
	Epigenetic changes	CIMP-high, MLH1 promoter methylation
	Metabolic reprogramming	Increased glycolysis, glutamine metabolism reliance
Clinical features	Histological features	Mucinous differentiation, poor differentiation
	Metastatic pattern	High rates of peritoneal and distant organ metastases
	Prognosis	Poor prognosis (median OS: 8-14 months)
	Chemoresistance	High resistance to conventional chemotherapy
Therapeutic response	Response to standard chemotherapy	Limited efficacy of fluoropyrimidine-based chemotherapy
	Effectiveness of BRAF inhibitors	Single-agent BRAF inhibitors show limited efficacy due to resistance
	Immunotherapy response	Effective in MSI-H, poor response in MSS
	Emerging targeted therapies	Combination therapies (BRAF+MEK+EGFR inhibitors) show improved outcomes
Molecular pathology	CpG island methylator phenotype (CIMP)	Frequent hypermethylation of tumor suppressor genes
	Microsatellite instability (MSI)	Commonly MSI-H but a significant subset is MSS
	Resistance mechanisms	Upregulation of alternative pathways (EGFR, PI3K/AKT, Wnt/β-catenin)

## Advances in treatment strategies

### 1. Targeted therapy and combination approaches

The development of targeted therapies for BRAFV600E-mutant CRC has made remarkable progress in recent years. Initial attempts to target the BRAF mutation using single-agent BRAF inhibitors, such as vemurafenib and dabrafenib, demonstrated limited efficacy due to the rapid onset of adaptive resistance, driven by upstream signaling pathway feedback activation. Consequently, combination therapies emerged as a key strategy to enhance clinical outcomes. The identification of co-occurring mutations, particularly in PIK3CA and PTEN pathways, has provided deeper insights into potential resistance mechanisms and guided the development of rational combination therapy approaches.

The landmark BEACON CRC clinical trial showed that the triple combination of encorafenib (BRAF inhibitor), binimetinib (MEK inhibitor), and cetuximab (EGFR inhibitor) significantly improved both progression-free survival and OS compared to standard chemotherapy regimens. Since then, this combination therapy has become the preferred second-line treatment for metastatic CRC patients harboring the BRAFV600E mutation. The rationale behind this combination therapy is to inhibit the MAPK pathway at multiple points, thereby preventing compensatory activation of parallel signaling pathways that contribute to resistance. Real-world evidence indicates that certain patient subgroups may derive greater therapeutic benefit from this regimen, especially those with minimal prior chemotherapy exposure^10)^.

Research is ongoing into combination therapies that target not only the MAPK pathway but also other critical oncogenic pathways. For instance, dual inhibition of BRAF and PI3K/AKT pathways has shown promising results in preclinical studies for overcoming MAPK inhibitor resistance. Furthermore, researchers are investigating the combination of BRAF inhibitors with immune checkpoint inhibitors (such as PD-1/PD-L1 blockers), particularly in microsatellite instability-high (MSI-H) tumors, which tend to show enhanced responsiveness to immunotherapy due to their high mutational burden^11)^. Ongoing clinical trials are evaluating novel adaptive therapy approaches, where drug combinations are dynamically adjusted based on real-time biomarker assessments to delay or prevent the development of resistance.

### 2. Immunotherapy

Immunotherapy has revolutionized the treatment landscape for multiple cancer types, including certain forms of CRC. However, the effectiveness of immunotherapy in BRAFV600E-mutated CRC remains complex and is largely dependent on the tumor’s microsatellite status. Tumors with high microsatellite instability (MSI-H) exhibit an elevated mutational burden, making them more responsive to immune checkpoint inhibitors such as pembrolizumab and nivolumab. In contrast, most BRAFV600E- mutant CRCs are MSS and show limited response to PD-1/PD-L1 blockade monotherapy. Recent preclinical studies have shown that targeting tumor-associated neutrophils and MDSCs in MSS tumors may enhance immune responsiveness, opening up new possibilities for combination immunotherapy approaches.

Recent therapeutic strategies have focused on enhancing the immunogenicity of MSS BRAFV600E- mutant CRCs. A promising strategy involves combining BRAF and MEK inhibitors, which can enhance tumor antigen expression and promote T-cell infiltration within the tumor microenvironment. Furthermore, the development of novel immunotherapeutic agents, such as bispecific T- cell engagers, which can simultaneously target both tumor cells and T cells, represents another promising strategy to enhance response rates in MSS tumors. Personalized cancer vaccines, designed to target specific neoantigens in BRAFV600E- mutant CRCs, are also being investigated as a means to enhance immune response.

### 3. Emerging Experimental Therapies

Ongoing clinical trials are evaluating next-generation BRAF inhibitors designed to enhance selectivity and minimize resistance development. One of these agents, belvarafenib, a type II RAF inhibitor, has shown promising preclinical activity and is currently being evaluated in combination with MEK and EGFR inhibitors. These next-generation therapies show promise for delivering more sustained responses, potentially overcoming several limitations of current BRAF inhibitors^12)^.

RNA-based therapeutics, particularly small interfering RNAs (siRNAs) designed to target oncogenic signaling pathways, represents an exciting frontier in cancer research. These RNA molecules can specifically downregulate the expression of key genes involved in BRAF-driven tumorigenesis, offering a novel approach to targeted therapy^13)^.

## Future directions

Despite significant progress in understanding and treating BRAFV600E-mutant CRC, this aggressive subtype remains a therapeutic challenge. There remains a critical need for more effective strategies to overcome therapy resistance and improve patient outcomes. The future of treating BRAFV600E-mutated CRC lies in integrating cutting-edge technologies, multi-omic approaches, novel therapeutic combinations, and precision medicine. Several key future directions that could lead to breakthroughs in the treatment and management of this challenging cancer subtype are outlined below. A deeper understanding of the tumor’s immune microenvironment and its role in disease progression will be crucial for developing next-generation therapeutic approaches.

### 1. Overcoming resistance to targeted therapies

BRAF inhibitor resistance remains a major challenge in treating BRAFV600E-mutant CRC. While combining BRAF inhibitors with MEK and EGFR inhibitors has shown clinical benefits, resistance mechanisms typically emerge, reducing the long-term effectiveness of these treatment regimens. Resistance mechanisms can be complex and involve multiple factors, including upregulation of alternative survival pathways, activation of upstream signaling feedback loops, and changes in the tumor microenvironment. Recent studies indicate that tumor metabolic adaptations, including enhanced oxidative phosphorylation and dysregulated lipid metabolism, may drive resistance mechanisms and represent potential therapeutic targets^14)^.

Future research should focus on elucidating the specific molecular pathways that drive resistance in BRAFV600E-mutant CRC. A promising research direction involves targeting the PI3K/AKT/mTOR pathway, which has been linked to MAPK inhibitor resistance across various cancer types, including CRC. Targeting this pathway in combination with BRAF inhibition could lead to a more sustained response by preventing the activation of parallel survival pathways. Furthermore, the role of epigenetic modifications in resistance remains an active field of research. Epigenetic reprogramming which encompasses DNA methylation, histone modifications, and chromatin remodeling can alter gene expression patterns and lead to treatment resistance. Understanding how BRAFV600E-mutant CRCs employ epigenetic modifications to evade treatment could lead to the development of novel drugs that reverse these changes and restore sensitivity to BRAF inhibitors. Combination therapies targeting both epigenetic regulators and oncogenic signaling pathways may provide a novel strategy to overcome resistance^15)^.

Recent preclinical studies have also highlighted how tumor-associated fibroblasts and immunosuppressive cells contribute to resistance against targeted therapies. The development of strategies to reshape the tumor microenvironment, such as combining BRAF inhibitors with antifibrotic agents or immune modulators, may enhance treatment efficacy and overcome drug resistance. A better understanding of tumor-stromal interactions will be crucial for developing therapies that target the microenvironment^16)^.

### 2. Expanding the role of circulating tumor DNA (ctDNA) in treatment monitoring and adaptation

The use of circulating tumor DNA (ctDNA) as a real-time biomarker is gaining traction as a non- invasive method to monitor treatment response and detect early resistance. ctDNA analysis can provide insights into tumor dynamics and clonal evolution, enabling clinicians to adjust therapy based on emerging resistance mutations. For instance, detecting RAS reactivation through ctDNA analysis during BRAF inhibitor therapy enables clinicians to switch to alternative treatment regimens before clinical progression occurs^17)^. Liquid biopsy techniques are being further refined to enhance the accuracy of minimal residual disease (MRD) detection, which could inform the selection of adjuvant treatment strategies.

Furthermore, monitoring MRD through ctDNA analysis could enhance post-treatment surveillance by identifying patients at high risk of relapse who might benefit from additional intervention. Future research should focus on optimizing ctDNA-based monitoring strategies and incorporating them into clinical decision-making to enhance patient outcomes. With advances in next-generation sequencing, ctDNA analysis could also enable real-time mutation tracking to guide precise treatment adjustments.

### 3. Immunotherapy and combination strategies

Immunotherapy has revolutionized the treatment landscape for multiple cancer types, including melanoma and non-small cell lung cancer, and has shown encouraging results in certain CRC subtypes. However, its role in BRAFV600E-mutant CRC remains less well understood. The vast majority of BRAFV600E-mutant CRCs are MSS, which typically results in low immunogenicity and limited response to immune checkpoint inhibitors, such as PD-1/PD-L1 blockade. Conversely, tumors with high microsatellite instability (MSI-H), which occur in a subset of BRAFV600E-mutated CRC cases, may show enhanced responsiveness to immune checkpoint inhibitors due to their elevated mutational burden^18)^. Novel therapeutic approaches, including the combination of immune checkpoint inhibitors with oncolytic viruses, are being investigated to enhance immune responses in MSS tumors.

Various combination strategies are being investigated to enhance immunotherapy effectiveness in BRAFV600E-mutant CRC. A promising strategy involves combining BRAF inhibition with immune checkpoint inhibitors, which could enhance tumor antigenicity and boost immune response. Furthermore, combining BRAF inhibitors with MEK inhibitors or other immunomodulatory agents could enhance the effectiveness of immunological therapies by preventing MAPK pathway feedback activation and promoting T-cell infiltration into the tumor microenvironment.

## Results and Discussion

This research is currently ongoing. This research represents a novel project that has never been conducted before in our laboratory. Therefore, we conceptualized the project, developed hypotheses, and conducted preliminary experiments during my studies abroad. This research is currently being carried out by my successor in the laboratory. BRAFV600E-mutant CRC represents a highly aggressive subtype of colorectal cancer that poses significant therapeutic challenges. Despite significant advances in targeted therapies and combination treatments, drug resistance remains a major barrier to long-term patient survival. Future research aimed at understanding the molecular underpinnings of this disease, identifying novel biomarkers, and developing innovative therapeutic approaches will be crucial for improving patient outcomes.

## Conclusion

BRAFV600E-mutant colorectal cancer represents a distinct and aggressive subtype characterized by poor prognosis and limited responsiveness to conventional therapies. In this study, we explored the molecular mechanisms underlying BRAFV600E- driven tumorigenesis and highlighted the therapeutic challenges associated with resistance to BRAF-targeted monotherapy. Our preliminary findings suggest that the combination of BRAF inhibitors with Wnt pathway inhibitors and epigenetic modifiers, such as DNMT inhibitors, may offer a promising therapeutic strategy.

The integration of Wnt signaling modulation and epigenetic reprogramming appears to enhance the sensitivity of BRAFV600E-mutant CRC cells to BRAF inhibition. Moreover, advances in circulating tumor DNA (ctDNA)-based monitoring provide a non-invasive approach for guiding therapeutic decisions and detecting emerging resistance in real time.

Although this research is ongoing, the insights gained lay a foundation for future investigations aimed at developing more effective and personalized treatment strategies for patients with BRAFV600E-mutant colorectal cancer. Continued efforts to elucidate the tumor microenvironment, resistance mechanisms, and molecular vulnerabilities will be crucial for improving clinical outcomes in this challenging cancer subtype.

As part of our future efforts, we plan to present our study titled “Investigation of BRAFV600E-Mutant Colorectal Cancer at Our Institution” at the 2025 Annual Meeting of the Japanese College of Surgeons. Furthermore, we aim to conduct a clinical study focused on the development of non-invasive monitoring methods using biomarkers in BRAFV600E-mutant colorectal cancer.

## Author contributions

All authors contributed equally to the manuscript.

## Conflicts of interest statement

The authors declare that there are no conflicts of interest.

## Availability of data and materials

The data and materials used in this study are available from the corresponding author upon reasonable request.
